# Polyclonal antibody-induced downregulation of HER1/EGFR and HER2 surpasses the effect of combinations of specific registered antibodies

**DOI:** 10.3389/fonc.2022.951267

**Published:** 2022-11-02

**Authors:** Gretchen Bergado-Báez, Narjara Gonzalez Suarez, Lisset Chao García, Dayana Pérez-Martínez, Diana Rosa Hernández-Fernández, Talia Fundora-Barrios, Antonio Rodríguez-Álvarez, Geidy Diana Díaz-Ordaz, Moshit Lindzen, Yosef Yarden, Belinda Sánchez-Ramírez

**Affiliations:** ^1^ Immunology and Immunotherapy Direction, Center of Molecular Immunology, Havana, Cuba; ^2^ Laboratoire d’Oncologie Moléculaire, Département de Chimie, Université du Québec à, Montréal, QC, Canada; ^3^ Department of Biological Regulation, Weizmann Institute of Science, Rehovot, Israel

**Keywords:** HER1, HER2, resistance, polyclonal antibodies, monoclonal antibodies, receptor degradation, cytotoxicity

## Abstract

**Background:**

Antitumor therapies targeting HER1/EGFR and HER2, such as monoclonal antibodies (MAbs) and tyrosine-kinase inhibitors (TKIs), have demonstrated a significant clinical benefit, but the emergence of resistance limits long-term efficacy. While secondary HER1 mutations confer tolerance to TKI, compensatory upregulation of HER2 drives resistance to anti-HER1 MAbs, which identifies MAb combinations targeting both receptors as an attractive therapeutic strategy. Nevertheless, toxicity hampers the clinical validation of this approach. Alternatively, cancer vaccines may induce antibodies directed against several antigens with less concern about induced toxicity.

**Methods:**

Polyclonal antibodies (PAbs) targeting HER1 and HER2 were induced in mice or rabbits through immunization. Recognition of different epitopes on targets by PAbs was validated by phage-display technology. Receptor downregulation was evaluated by flow cytometry, immunofluorescence, and Western blot. MTT assays assessed cytotoxicity, while the antitumor effect of PAbs was assayed in nude mice.

**Results:**

PAbs promoted degradation of HER1 and HER2 regarding clinical MAbs or their combinations. As a result, inhibition of cytotoxicity on tumor cell lines was improved, even in the presence of oncogenic mutations in HER1, as well as in cetuximab-insensitive cells. Accordingly, the antitumor effect of vaccination-induced PAbs was observed in lung tumor lines representative of sensitivity or resistance to HER1 targeting therapies.

**Conclusions:**

Immunization against HER1 and HER2 receptors offers an alternative to passive administration of combinations of MAbs, since vaccination-induced PAbs promote the downregulation of both receptors and they have a higher impact on the survival of tumor cells.

## 1 Introduction

In the past decades, increasing preclinical and clinical lines of evidence have highlighted the role of the epidermal growth factor receptor (EGFR/HER1), and its prominent partner HER2, as oncogenes and targets for antitumor therapies (1). Monoclonal antibodies (MAbs) targeting HER1 like cetuximab (2), panitumumab (3), and nimotuzumab (4), as well as tyrosine kinase inhibitors (TKIs) like erlotinib (5) have demonstrated a remarkable clinical benefit and are approved for the treatment of lung, colorectal, or head/neck cancer (6). As for HER2, specific MAbs trastuzumab (7) and pertuzumab (8) revolutionized the treatment of HER2-positive breast cancer (9). While effector mechanisms sustaining the efficacy of TKI include proliferation arrest and apoptosis induction (10), MAbs rely on Fab-dependent mechanisms like inhibition of homo- or heterodimer formation and endocytosis of the targets, along with Fc-dependent mechanisms that involve the recruitment of innate immune effectors (11).

Despite the clinical benefit of these therapies, patient relapse is often associated with the emergence of tumor-resistant variants (12). The most common resistance-driving mutation in response to HER1-targeting TKIs is a threonine–methionine amino acid substitution at position 790 (T790M) of HER1, which mediates tolerance to first-generation TKIs like gefitinib and erlotinib by increasing the affinity for ATP (6). Also, HER1-exon 19 deletions are the most recurrent activating mutations in advanced non-small cell lung cancer (NSCLC) (13). In contrast, resistance to MAbs is often associated to bypass signaling driven by genomic alterations in downstream signaling molecules like KRAS and PI3KCA, which activate downstream pathways ERK1/2 and PI3K-AKT, respectively (14). As an alternative, compensatory upregulation of additional HER family members like HER2 or related receptors like MET in response to chronic treatment with cetuximab enables bypass signaling and tumor recurrence (15). Hereafter, sustained inhibition of several HER receptors has been proposed as a therapeutic alternative to avoid resistance emergence (16). In support of this hypothesis, preclinical studies have demonstrated the superiority of the combination of two registered MAbs, cetuximab and trastuzumab, with regard to single molecules (17) or standard chemotherapy (18). Unfortunately, clinical validation of this approach has been limited by the increase of toxicity (19, 20).

On the other hand, MAbs-induced downregulation of the targets is counteracted by endosomal escape and recycling following endocytosis. In this regard, oligoclonal antibody cocktails have been developed to mimic polyclonal antibodies’ (PAbs) response (as induced by infection or vaccination) and to improve their therapeutic effects (21). However, this approach has the drawbacks of enhanced toxicity and limited polyclonality, which could be heightened by cancer vaccines that are characterized by a remarkable safety profile (22). Afterwards, endogenous PAbs induced by active immunization could be an alternative to oligoclonal mixtures of MAbs. In a previous report, we described a protein-subunit vaccine candidate that induced PAbs simultaneously targeting HER1 and HER2 in mice, which were able to promote the degradation of both receptors expressed on H292 tumor cells (23). However, this model is highly sensitive to specific registered anti-HER1 TKIs and MAbs, and is, thereafter, representative of a clinical scenario where this vaccine candidate could be less useful. Elucidation of a suitable, more demanding niche of patients that cannot benefit from the administration of MAbs, where endogenous PAbs could make a difference, is necessary. The present study aims to compare vaccination-induced PAbs with combinations of registered MAbs attending to their differential impact on HER1 and HER2 downregulation, subsequent inhibition of signaling through these receptors, and impairment of cell viability in tumor lines that are models of resistance to HER1-targeting therapies.

## 2 Materials and methods

### 2.1 Animals

Female BALB/c mice aged 8–12 weeks old, female Nu/Nu nude mice aged 6 weeks old, and male New Zealand (NZD) rabbits were acquired from the National Center for Laboratory Animals Production (CENPALAB, Havana, Cuba). All mice were kept under pathogen-free conditions. Animal experiments conducted were approved by the Center of Molecular Immunology’s Institutional Animal Care and Use Committee (CIM, Havana, Cuba).

### 2.2 Generation of PAbs targeting HER1 and HER2

BALB/c mice (*n* = 40) were subcutaneously immunized four times biweekly with a final volume of 400 µl per mice (divided into two injection sites, receiving 200 µl on each site). The vaccine candidate formulation included 400 µg of human variants of HER1-extracellular domain (HER1-ECD) and HER2-ECD generated as previously described (23). The adjuvant used in the formulations was VSSP (very small-sized proteoliposomes, 200 µg per injection per mouse) derived from the outer membrane of *Neisseria meningitidis* that has been proven to act as an immune system modulator by reducing the regulatory function of myeloid-derived suppressor cells (MDSCs) (24). On day 56, mineral oil was administered into the peritoneal cavity of mice, and 3 days later, X63 myeloma cells (10^6^ per mouse, diluted in PBS, considering a final volume of 100 µl per mouse) were inoculated to induce ascites formation. Ascites was clarified and IgGs were captured with Protein A. Finally, specific PAbs were isolated by immunoaffinity. Control antibodies were obtained from non-immunized mice and captured by Protein A chromatography after clarification.

Alternatively, NZD rabbits (*n* = 3) were immunized four times as previously described. A control group was included to obtain irrelevant PAbs. On day 56, both immunized and control groups of animals were bled, and sera were extracted from blood and subsequently dialyzed against sodium acetate 0.02 M buffer, containing 0.2 M of sodium chloride, after which IgG isotype antibodies were purified by Protein A chromatography.

### 2.3 Monoclonal antibodies and reagents

Nimotuzumab (TheraCIM, hR3) was obtained from CIMAB S.A., Cuba. Trastuzumab (Herceptin), pertuzumab (Perjeta), cetuximab (Erbitux), and panitumumab (Vectibix) were obtained from commercial sources (Roche, Genentech, Merck KGaA, and Amgen, respectively). D1 fusion protein comprising an anti-HER-1 domain IV scFv fused to a human Fc domain was produced at the Protein Engineering Laboratory of the Center of Molecular Immunology (Cuba).

The fluorescent antibodies used for FACS anti-EGFR-Alexa Fluor 488 (#352108) and HER2-APC (#324408) were obtained from BioLegend. Quantitative ELISA systems used for detection of HER1 (DEGFR0) or HER2 (DHER20) were obtained from R&D Biosystems. For Western blot assays, antibodies targeting HER1 (#4267S), phosphorylated HER1 (Y1068, #2234S), HER2 (#4290S), phosphorylated HER2 (Y1221/1222, #2249S), STAT3 (#9132S), phosphorylated STAT3 (Y705, #9145S), AKT1 (#2938S), phosphorylated Akt (S473, #4060S), ERK1/2 (#4695S), and phosphorylated ERK1/2 (Y202/T204, #9101S) were acquired from Cell Signaling Technologies; an antibody targeting GAPDH (#MAB374) was obtained from Millipore (dilution 1:15,000). Antibodies targeting HER1 and HER2 acquired from Cell Signaling were also used in immunofluorescence assays. The TKI AG1478 (Tyrophostin AG-1478, #T4182-5MG) and lapatinib (#CDS022971-25MG) were purchased from Sigma-Aldrich, while osimertinib (Tagrisso^®^) was gifted by AstraZeneca to Prof. Yosef Yarden’s lab. The 3-(4,5-Dimethylthiazol-2-yl)-2,5-diphenyl tetrazolium bromide (MTT, #M5655) reagent was obtained from Sigma-Aldrich. Growth factors were from PeproTechAsia (Israel).

### 2.4 Cell lines and culture conditions

Eight NSCLC cell lines were used in our study. H292, H3255, H1975, PC9, A549, and H460 were obtained from the American Type Tissue Culture Collection (ATCC), as well as SKBR3 derived from a breast adenocarcinoma. The lung adenocarcinoma H125 was gently donated by the Molecular Biology Department of MPI (Germany). The erlotinib-resistant PC9ER cell line was obtained by the Department of Biological Regulation of the Weizmann Institute of Sciences (Israel) (25). Cell lines were maintained in basal growth media (RPMI in the case of H292, H3255, H1975, PC9, and PC9ER cells, or DMEM for H125, A549, and H460 cells) acquired from Gibco and supplemented with 10% fetal calf serum (FCS; Gibco) and penicillin/streptomycin (100 µg/ml). SKBR3 cells were grown in McCoy’s 5A culture medium supplemented with 15% FCS and antibiotics.

### 2.5 Western blotting

Cells were grown under specified conditions, treated or stimulated as indicated, washed twice with ice-cold phosphate buffered saline (PBS), and scraped into lysis buffer [50 mM Hepes (pH 7.5), 10% glycerol, 150 mM NaCl, 1% Triton X-100, 1 mM EDTA,1 mM EGTA, 10 mM NaF, 0.1 mM Na_3_VO_4_, and a complete protease inhibitor cocktail]. Next, lysates were centrifuged at 14,000*g* for 15 min at 4°C and supernatants were collected for further procedures. Proteins were separated using gel electrophoresis and transferred onto nitrocellulose membranes. After blocking, membranes were incubated overnight with the indicated primary antibodies, followed by incubation with horseradish peroxidase-conjugated secondary antibodies for 1 h, and treatment with Clarity™ Western ECL Blotting Substrates (Bio-Rad). ECL signals were detected using the ChemiDoc™ Imaging System (Bio-Rad) and images were acquired using the ImageLab software.

### 2.6 ELISAs

#### 2.6.1 Phage-display recognition of HER1/HER2 subdomains

The genes coding for the subdomains of HER-1 and HER-2 ECDs (flanked by *Not*I and *Sal*I restriction sites) were amplified by PCR and cloned into pc89-c-myc phagemid vector, inserted near the 5’ end of the M13 PVIII-coding gene. The sequences of every insert were confirmed by Macrogen, Korea. Phage particles displaying the domains were rescued with M13KO7 helper phage following established procedures (26). Aiming to assess the recognition of phage-displayed HER domains, polyvinyl chloride microtiter plates were coated overnight at 4°C with 10 μg/ml of MAbs or PAbs diluted in PBS. Plates were blocked with 4% (w:v) milk in PBS (M-PBS). Purified phages displaying the subdomains of HER1 or HER2 (diluted in M-PBS) were added to the plates. Next, an anti-M13 MAb conjugated to horseradish peroxidase (HRP, GE Healthcare) appropriately diluted in M-PBS was added. Substrate solution (500 μg/ml ortho-phenylenediamine and 0.015% hydrogen peroxide in 0.1 mol/L citrate-phosphate buffer, pH 5.0) was added and the reaction was stopped after 15 min with sulfuric acid (2.5 mol/L). The absorbance at 490 nm was determined with a microplate reader (Dialab GmbH ELx808). Incubations were performed at room temperature for 1 h. Following incubation steps, plates were washed several times with PBS/0.05% Tween 20.

The amounts of different phage-displayed domains within each experiment were normalized measuring recognition of c-myc tag (fused to all displayed proteins) on 9E10-coated microtiter plates, using established procedures (26). A standard curve of phages displaying c-myc alone was taken as reference, assuming the presence of 100 arbitrary display units/ml in the undiluted preparation. The relative display levels of HER domains in each preparation were calculated by interpolation from the standard curve.

#### 2.6.2 Inhibition of HER1 or HER2 recognition by specific MAbs

Microtiter plates (High binding, Costar) were coated with 5 μg/ml of HER1-ECD or HER2-ECD diluted in carbonate buffer, 0.1 M, pH 9.6, and incubated overnight at 4°C. Plates were then blocked with 5% FCS in PBS/0.05% Tween 20 (blocking buffer). Next, plates were incubated with the PAbs contained within immune sera diluted in blocking buffer, ranging from 1/25 to 1/25,600. Nimotuzumab (160 ng/ml), cetuximab (40 ng/ml), panitumumab (40 ng/ml), or D1 (40 ng/ml) was further added to HER1-coated plates, while trastuzumab (40 ng/ml) or pertuzumab (80 ng/ml) was added (40 ng/ml) to HER2-coated plates. Afterwards, plates were incubated with an alkaline phosphatase (AP)-conjugated goat anti-human IgG antibody (Sigma, #A3187) diluted in blocking buffer (1/1,000). Following the addition of p-nitrophenylphosphate (1 mg/ml) (Sigma, #N9389) diluted in diethanolamine buffer, pH 9.8, absorbance at 405 nm was detected in a microplate reader (Dialab GmbH ELx808). Incubation steps were performed at 37°C for 1 h. Between incubations, plates were washed three times with PBS/0.05% Tween 20.

#### 2.6.3 Quantitative detection of HER1/HER2

Cells were grown under specified conditions, treated as indicated, washed twice with cold PBS, and lysed. HER1 or HER2 were detected within 10 µg of total proteins using commercial ELISA systems (R&D). Assay development and analysis were performed following the manufacturer’s instructions.

### 2.7 Determination of receptor endocytosis by flow cytometry

To determine surface receptor abundance, H292, H1975, or PC9ER cells were seeded in six-well plates (1.0 × 10^6^ per well). The next day, media were replaced and cells were treated as indicated for an additional 24 h. Thereafter, cells were harvested with trypsin and washed twice in PBS. Next, cells were washed in acidic buffer (glycine–HCl 100 mM, pH 3.0) and incubated with fluorescent antibodies, following the manufacturer’s instructions (5 µl per million cells in 100 µl of staining volume). To exclude death cells from the analysis, cells were stained with DAPI. Surface signals were analyzed using a BD FACS Aria Fusion cytometer.

### 2.8 Immunofluorescence assays

H292 or H1975 cells were grown on autoclaved coverslips in 12-well plates (1.0 × 10^5^ per well) and treated as indicated for 24 h. Next, cells were washed in acidic buffer (glycine-HCl 100 mM, pH 3.0), washed three times with PBS, and fixed in paraformaldehyde 4% for 15 min, followed by permeabilization in 0.3% Triton X-100 for 10 min. Cells were then incubated for 1 h with 3% albumin in PBS-T, followed by incubation with a primary antibody in PBS-T containing 1% albumin (overnight at 4°C), washed in PBS-T, and incubated with a fluorescently labeled secondary antibody (Alexa Fluor 555) and DAPI for 1 h at room temperature. Images were captured using a Zeiss confocal microscope (40× magnification) and processed using the ImageJ software.

### 2.9 Viability assays

Cell viability was assessed by MTT assay. Cells were plated in 96-well plates as follows: 5 × 10^3^ cells/well in the case of H292, H125, H1975, A549, H460, and SKBR3 cell lines; 3 × 10^3^ cells/well for PC9 and PC9ER cell lines; and 20 × 10^3^ cells/well for the H3255 cell line. All reached a 60%–70% confluence 24 h later. The medium was replaced and cells were treated as indicated in triplicates. After 72 h, the MTT reagent was added to the cells (1 mg/ml), and 2 h later, the formazan crystals were dissolved in DMSO. Absorbance was measured at 540 nm, and background at 630 nm was subtracted. All experiments were performed in growth medium containing 1% fetal calf serum (FCS).

### 2.10 Antitumor assays

H292 (10^6^per mouse) or PC9 cells (3 × 10^6^per mouse) were subcutaneously injected in the right flanks of 6-week-old female Nu/Nu nude mice (NU-*Foxn1^nu^*). Once the length and width of the tumors reached 3 × 3 mm, mice were randomized into two groups and treated as indicated. Irrelevant vaccination-induced PAbs were administered twice a week using intraperitoneal injection at a final dose of l mg of total IgG/mouse/injection. Tumors were measured with a caliper. Tumor volume was calculated by using the formula 3.14 × shortest diameter × (longest diameter)^2^ × 1/6.

### 2.11 Statistical and data analyses

GraphPad Prism 7.0 software was used for data representation. Statistical analysis was performed using IBM SPSS Statistics 19 and GraphPad Prism 7.0 programs. ImageLab software was used for image acquisition corresponding to the detection of luminescence in Western blot assays. Densitometric analysis of the blots and quantitative analysis of the images from immunofluorescence were developed using ImageJ 2.1 software. Flow cytometry analysis was performed using FlowJo 7.6 software. In competitive ELISAs, the serum dilutions were log transformed, and data were adjusted to a log (PAbs dilution) *vs*. normalized response with variable slope nonlinear regression. Normality was evaluated by Shapiro–Wilk test, and variance homogeneity was analyzed using Levene or Brown–Forsythe test. Statistical differences among groups’ media were analyzed using one-way ANOVA followed by Tukey-multiple comparisons test. Alternatively, when variances were not homogeneous, or when normal distribution was not observed even after scale transformation, Kruskal–Wallis test and Games–Howell post-test were used, as well as Mann–Whitney *U* test. In graphic representations, significant differences were highlighted with asterisks: *p < 0.05, **p < 0.01, ***p < 0.001, and ****p < 0.0001.

## 3 Results

### 3.1 PAbs recognize different subdomains of HER1 and HER2

**Figure 1:**
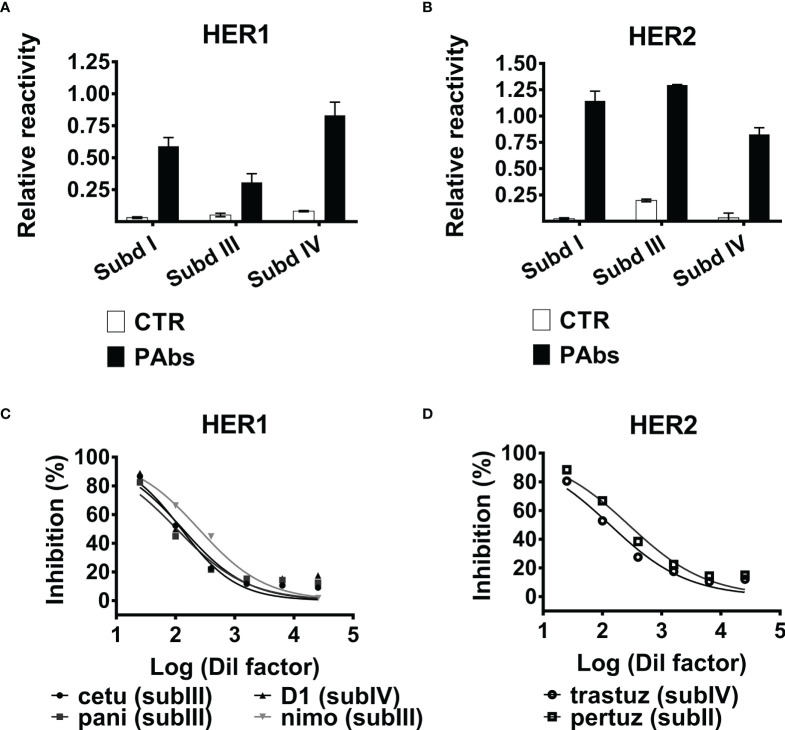
Recognition of HER1/HER2 subdomains by the PAbs and inhibition of MAbs binding. Recognition of **(A)** HER1 or **(B)** HER2 subdomains I, III, and IV expressed on phages by the PAbs (10 µg/ml) assessed by ELISA. Samples were normalized against the absorbance obtained for the recognition of 9E10MAb. PAbs from non-immunized mice (CTR PAbs) were included as control. Graphs represent the increase of the absorbance at 490 nm corresponding to the recognition of each subdomain by specific or control PAbs, normalized considering the absorbance of the 9E10 control and expressed as relative reactivity. **(C)** HER1-ECD-coated plates were incubated with dilutions of PAbs-containing immune sera; next, the binding capacity of MAbs was evaluated by ELISA. Graphs represent the inhibition of HER1-ECD-targeting cetuximab (cetu, black circle), panitumumab (pani, black square), nimotuzumab (nimo, gray inverted triangle), and D1 (black triangle). Inhibition was expressed as percentage considering binding of the MAbs in the absence of PAbs as maximum reactivity control, and data were adjusted to a non-linear “response *vs*. log (inhibitor)” regression with variable slope. **(D)** Likewise, it was evaluated the inhibition of the binding of HER2-ECD recognizing trastuzumab (trastuz, open circle) and pertuzumab (pertuz, open square), mediated by the PAbs. A representative experiment of three performed is shown.

Immunization of BALB/c mice with truncated fragments of the extracellular domains (ECDs) of HER1 and HER2 has the potential of inducing antibodies against multiple epitopes within each antigen. In order to dissect the whole PAbs’ response induced by our bispecific candidate into partial reactivities against some individual subdomains of the targets, subdomains I, III, and IV of both HER1 and HER2 were individually displayed as PVIII fusion proteins on filamentous phage, following a described methodology (26). As observed in **Figures 1A** **, B**, immunization-induced PAbs reacted against the three subdomains of both receptors, unlike irrelevant PAbs obtained from naive mice. This result indicates that, as predicted, antibody response specific for each receptor induced by immunization is polyclonal. Folding of Cys-rich subdomain II of HER receptors, which contains the “dimerization arm” of HER receptors, is drastically affected when expressed individually in eukaryotic expression systems (27); thus, recognition of subdomain II of both antigens was limited.

Additionally, it was observed that recognition of soluble ECDs of HER1 and HER2 by the PAbs inhibited further binding of MAbs targeting different subdomains of both HER1 (panitumumab, cetuximab, nimotuzumab, or D1) and HER2 (pertuzumab and trastuzumab) in a dose-dependent manner (**Figures 1C** **, D**), supporting the idea of a multi-subdomain recognition of both receptors by the PAbs. Of note, panitumumab, cetuximab, and nimotuzumab recognize different (partially overlapped) epitopes within subdomain III of HER1 (28). It was also observed that inhibition of the three MAbs targeting subdomain III of HER1 was similar regardless of their differential affinities (*K*_D_ panitumumab = 5 × 10^−11^; *K*_D_ cetuximab = 0.39 × 10^−9^; *K*_D_ nimotuzumab = 10^−8^) (29, 30). Then, the binding strength of the PAbs (avidity) and some of these MAbs (affinity) to the targets was compared in the presence of a chaotropic agent by ELISA, as previously described (31). Interestingly, the binding strength of HER1-specific PAbs was lower than cetuximab but higher than nimotuzumab, whereas HER2-specific PAbs were inferior than trastuzumab or pertuzumab. These lines of evidence suggestthat the avidity of the PAbs recognizing HER1 and HER2 could be lower than high-affinity MAbs like cetuximab and trastuzumab, respectively (**Figure S1**); still, antigen recognition by these MAbs is prevented by these lines of evidence pointing to the multi-epitope recognition of the targets achieved by the PAbs.

### 3.2 Degradation of HER1 and HER2 is potentiated by polyclonal recognition

**Figure 2:**
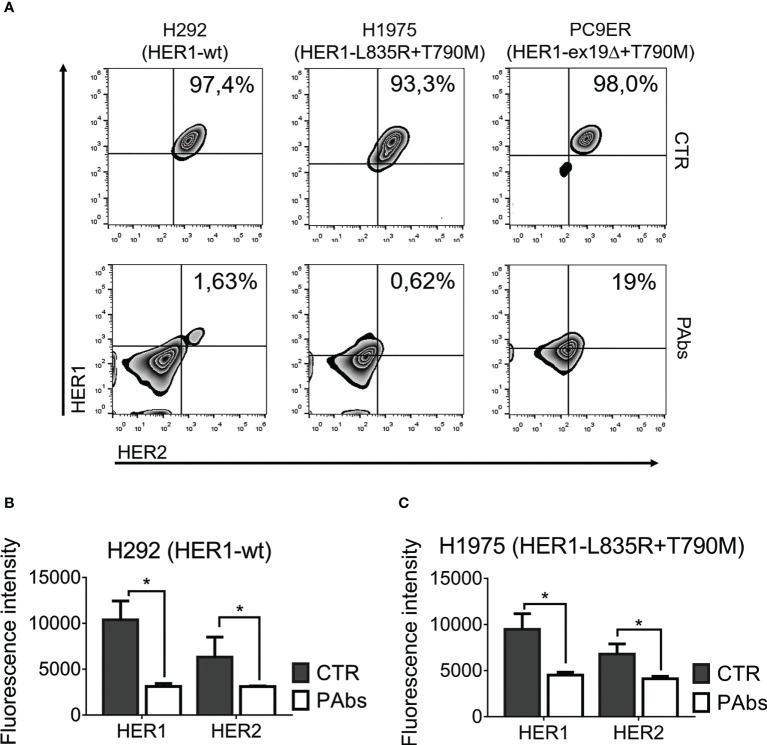
Promotion of the endocytosis and degradation of HER1 and HER2 by the PAbs. **(A)** H292, H1975, and PC9ER cells were treated for 24 h with PAbs-containing immune sera or pre-immune sera as negative control (1/100). After washing with acidic buffer (glycine 100 mM, pH 3.0), cells were incubated with antibodies against HER1 and HER2 tagged to fluorophores, and surface expression of the receptors was analyzed using flow cytometry. Death cells were excluded with DAPI. Density plots show the distribution of the cell population according to recognition of HER1 (*y*-axis) and HER2 (*x*-axis), and the percentage of cells expressing both receptors is specified. Data are representative of two independent experiments. **(B)** H292 and **(C)** H1975 cells were seeded in coverslips and treated as in **(A)** for 24 h. Cells were washed in acidic buffer, fixed in paraformaldehyde (4%), and incubated with specific primary antibodies, followed by an Alexa Fluor 555‐conjugated secondary antibody. Images were captured using a confocal microscope (40× magnification) and quantification was performed using ImageJ software. In the graphs, data are means ± SD of triplicates in a representative experiment of three conducted. Means were compared using Kruskal–Wallis test followed by a Games–Howell post-test. Significant differences among control and PAbs-treated cells are represented *p < 0.05.

Initially, the impact of the PAbs on receptors’ surface and total expression was evaluated (**Figure 2**). This analysis was performed in NSCLC cell lines like H292, overexpressing wild-type HER1 with moderate HER2 expression. Additionally, we included cell lines expressing mutated variants of HER1 that confer resistance to gefitinib/erlotinib, favor recycling over degradation following internalization of the receptor, and enhance its catalytic and oncogenic activity (32, 33). H1975 cells harbor the L858R/T790M double mutation in HER1 and PC9ER cells, carrying the exon 19 deletion (exon19Δ) acquired *in vitro* by chronic exposure to erlotinib (34). As observed, the PAbs substantially reduced the detection of HER1 and HER2 on the surface of H292, H1975, or PC9ER cells (**Figure 2A**). In tumor cells, HER1 and HER2 are preferentially located at the cell surface, but they can be also detected intracellularly (even in the nucleus, where they act as transcription factors) according to their trafficking and recycling mechanism (35). Then, we analyzed surface and intracellular expression of these receptors by immunofluorescence analysis of permeabilized H292 and H1975 cells, which evidenced that endocytosis of HER1 and HER2 conducted to their degradation (**Figures 2B** **, C** and **Figure S2**). Degradation of HER2 was apparently more pronounced, in comparison with HER1. This could be due to the expression levels of HER2 in H292 cells, which are lower than HER1 by almost one order of magnitude (23).

Nevertheless, our study focuses on the comparative evaluation of functional attributes of vaccination-induced PAbs with combinations of paired registered MAbs targeting HER1 and HER2. The combination of cetuximab and trastuzumab was selected based on preclinical evidence supporting the synergistic effect of their concomitant use (18). Also, HER1-specific nimotuzumab was characterized by 10 times lower affinity than cetuximab (29), and a notable safety profile (36) was evaluated in combination with trastuzumab.

**Figure 3:**
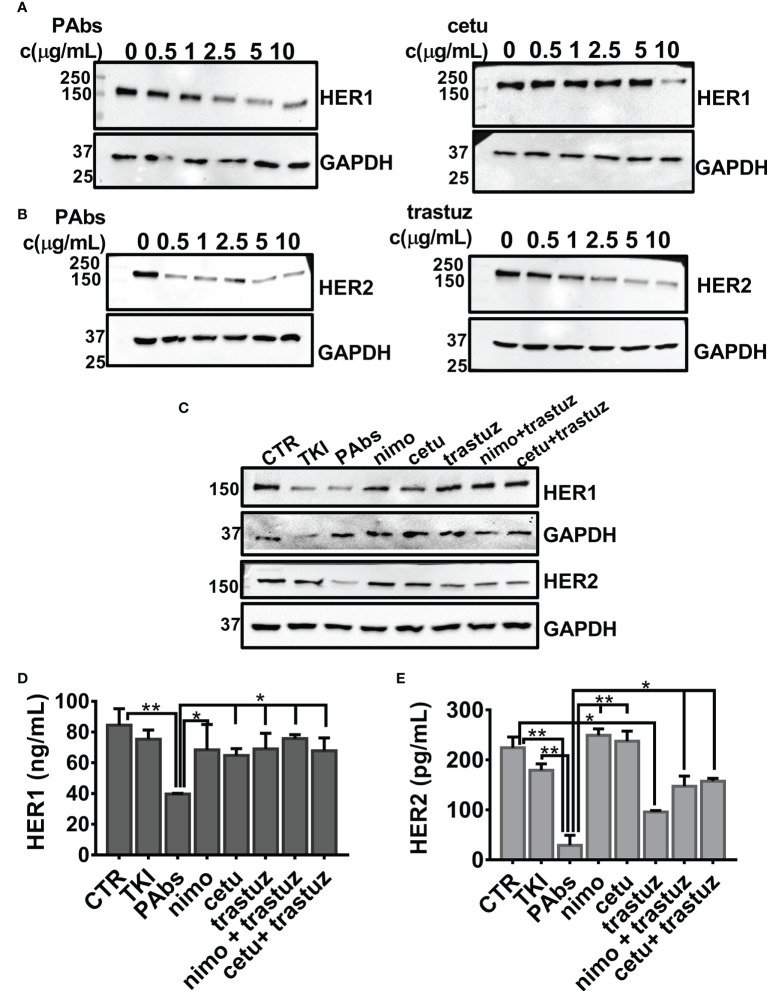
Comparative evaluation of the degradation of HER1 and HER2 in H292 cells expressing wild-type HER1. For dose-curve assessment, H292 cells were treated for 24 h with increasing concentrations (0, 0.5, 1, 2.5, 5, and 10 µg/ml) of the PAbs, cetuximab (cetu), or trastuzumab (trastuz). Thereafter, cells were washed with cold saline and lysed. Next, **(A)** HER1 (3 µg of total lysates) and **(B)** HER2 (30 µg of total lysates) expression levels were analyzed by Western blot. Detection of GAPDH was included as loading control for individual membranes. **(C)** For point-dose evaluation, H292 cells were treated for 24 h with the PAbs (10 µg/ml), or with combinations of cetuximab (cetu, 10 µg/ml) or nimotuzumab (nimo, 10 µg/ml) with trastuzumab (trastuz, 1 µg/ml). Unspecific PAbs (CTR, 10 µg/ml), single MAbs, or TKI AG1478 (10 µM) was included as controls. As previously described, expression levels of HER1 (3 µg of total lysates) and HER2 (30 µg of total lysates) were analyzed by Western blot, including GAPDH detection as loading control. Alternatively, quantitative ELISA was performed to measure **(D)** HER1 and **(E)** HER2 in the lysates of H292 cells treated as in **(C)**, following the manufacturer’s instructions. In the graphs, data are means ± SD corresponding to triplicates. A representative experiment of three performed is shown. Group means were compared using Kruskal–Wallis test followed by a Games–Howell post-test. Significant differences among PAbs and control, MAbs or its combinations are represented *p < 0.05; **p < 0.01. MWM—molecular weight marker.

A dose-dependent evaluation of PAbs-induced downregulation of HER1 and HER2 was compared to the magnitude reached by high-affinity cetuximab or trastuzumab in the NSCLC cell line H292 expressing wild-type variants of both receptors. After 24 h of incubation, dose-curve assessment of the expression levels of HER1 and HER2 evidenced that the PAbs enhanced the downregulation of HER1 at four times lower concentrations than cetuximab (2.5 µg/ml of PAbs *vs*. 10 µg/ml of cetuximab) (**Figure 3A**). Likewise, HER2 degradation was observed at five times lower dose of the PAbs (0.5 µg/ml) with regard to trastuzumab (2.5 µg/ml) (**Figure 3B**). In additional dose-point evaluations, PAbs (10 µg/ml) were compared to 10 µg/ml nimotuzumab/cetuximab and 1 µg/ml trastuzumab, considering equivalent recognition of the targets (**Figure S3**). Of note, the concentration used for anti-HER1 MAbs (that is, 10 µg/ml for cetuximab and nimotuzumab) was consistent with previous literature reports where these MAbs were used *in vitro* (34, 37). Likewise, the concentration of anti-HER2 MAb, trastuzumab, agreed with additional literature reports (38, 39). As before, PAbs-induced degradation of HER1 and HER2 exceeded HER1-specific or HER2-specific MAbs and their combinations in H292 cells, as evidenced by immunoblotting (**Figure 3C**) and quantitative ELISA (**Figures 3D** **, E**).

These lines of evidence were complemented in NSCLC cell lines overexpressing mutated variants of HER1, like H1975 cells (**Figures S4A, C, E**) and PC9ER cells (**Figures S4B, D, F**). Once again, downregulation of the targets was more drastic for PAbs-treated cells despite the presence of mutations in the HER1-tyrosine-kinase domain. These results support the hypothesis that degradation of the targeted receptors is enhanced following multi-epitope recognition, as attained by the PAbs.

In addition to the evaluation of the impact of PAbs and MAbs combinations in HER1 and HER2 expression, we also aimed to compare their effect on the activation and downsignaling through these receptors. Since H292 cells overexpress wild-type HER1, ligand-induced phosphorylation of this receptor and HER2 transactivation can be evaluated after stimulation with specific ligands. After 1 h of pre-incubation, PAbs inhibited both HER1 and HER2 phosphorylation induced by low-affinity amphiregulin (**Figure S5A**) and epiregulin (**Figure S5B**) or high-affinity EGF (**Figure S5C**) and HB-EGF (**Figure S5D**) in comparison with unspecific PAbs (CTR). While the impact of PAbs was similar to the combination of the high-affinity MAbs cetuximab–trastuzumab for low-affinity ligands (amphiregulin and epiregulin), their ability to neutralize high-affinity ligands (EGF and HB-EGF) was less pronounced. Inhibition of downsignaling proteins related to the major signaling cascades recruited by HER1 and HER2 (STAT3, ERK1/2, and Akt) was also characterized in H292 cells expressing wild-type HER1 (**Figure S6A**), H1975 cells expressing mutated HER1 (**Figure S6B**), and A549 bearing the KRas–G12S substitution (**Figure S6C**). As observed, inhibition of STAT3 by PAbs was comparable to TKI control and superior to MAbs in the three scenarios. The impact on Akt and ERK1/2 phosphorylation was less extensive in the case of HER1-mutated H1975 cells, which could be related to compensatory receptors that are not affected by HER1/HER2-targeting PAbs. In A549 cells expressing mutated KRas, PAbs were able to block the phosphorylation of Akt (in addition to STAT3) though inhibition of ERK1/2 was not achieved.

### 3.3 PAbs affect the cell viability in NSCLC cell lines resistant to anti-HER1 therapies where combinations of MAbs are not effective, and elicit an antitumor effect in models of sensitivity or resistance to HER1-targeting therapies

**Figure 4:**
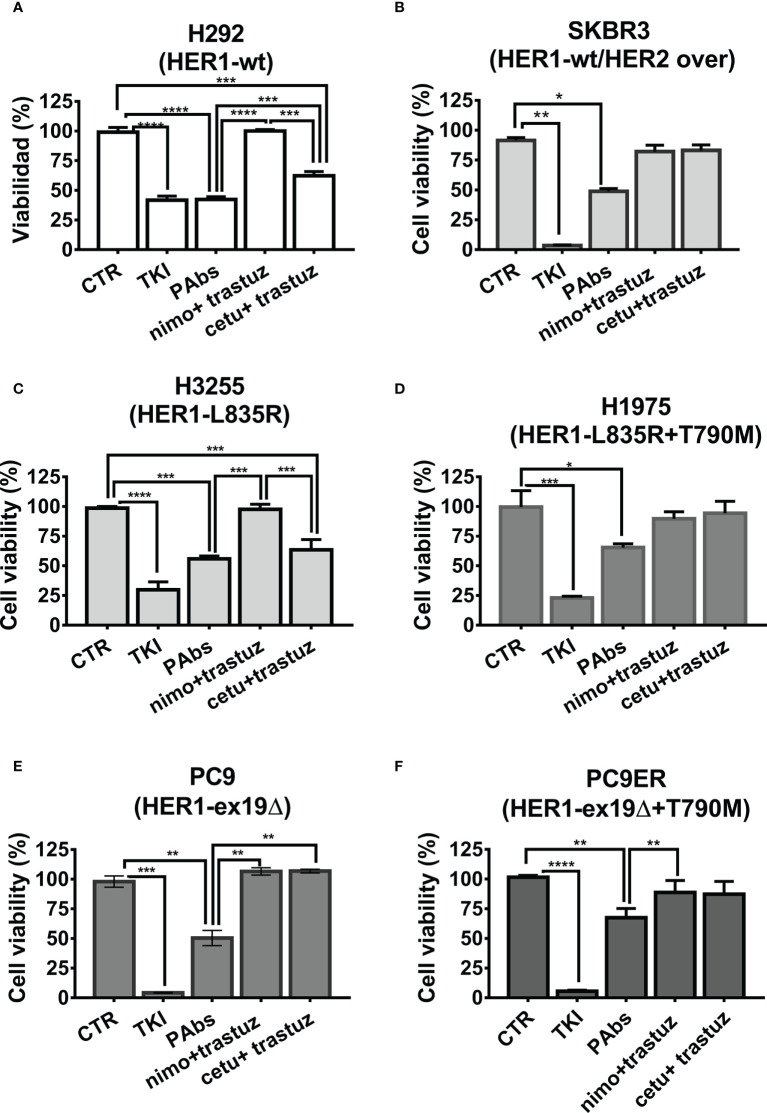
Inhibition of tumor cell viability. Cells were treated with PAbs (10 µg/ml) or with the combination of cetuximab (cetu, 10 µg/ml) or nimotuzumab (nimo, 10 µg/ml) with trastuzumab (trastuz, 1 µg/ml). Unspecific PAbs (CTR, 10 µg/ml) or specific TKI AG1478 (in the case of H292 and A549 cells, 10 µM), lapatinib (in the case of SKBR3 20 µM), or osimertinib (for HER1-mutated lines cells,1 µM) was included as negative and positive controls, respectively. After 72 h of treatment, the viability of **(A)** H292, **(B)** SKBR3, **(C)** H3255 (L835R), **(D)** H1975 (L835R/T790M), **(E)** PC9 (exon19 deletion), and **(F)** PC9ER cells (exon 19 deletion and T790M mutation) was evaluated by MTT. In the graphs, data are means ± SD of triplicates in one experiment representative of at least two conducted, for each cell line. Differences among means, when normality and variance homogeneity were confirmed, were analyzed using one-way ANOVA, and Tukey test was used for multiple comparisons. Alternatively, non-parametric Kruskal–Wallis test was conducted, followed by Games–Howell post-test. Significant differences among negative control PAbs and the treatments as well as among specific PAbs and MAbs combinations are represented *p < 0.05; **p < 0.01; ***p < 0.001; ****p < 0.0001.

Next, we compared the differential impact on proliferation and survival of PAbs and MAbs combinations in a panel of NSCLC harboring different mutations in the catalytic domain of HER1. It was observed that H292 cells’ viability was affected by both PAbs and the combination of cetuximab–trastuzumab (**Figure 4A**). Moreover, dose-curve evaluation showed that PAbs were cytotoxic at lower doses than the combination of cetuximab and trastuzumab in this model (**Figure S7A**). Cytotoxicity was also compared in SKBR3 cells, with overexpression of wild-type HER2 and mild expression of wild-type HER1 (**Figure 4B**) and where PAbs induced a higher reduction of the cell viability than MAbs at the evaluated treatment conditions.

**Figure 5:**
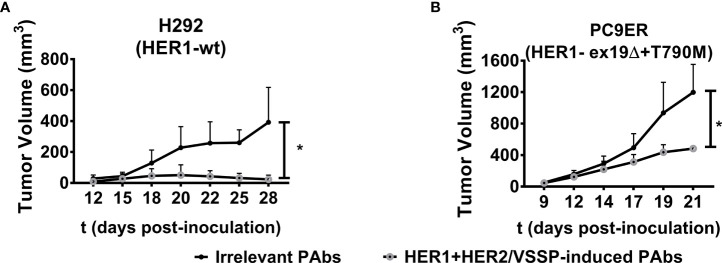
Inhibition of tumor growth. H292 (10^6^ per mouse) or PC9 cells (3 × 10^6^ per mouse) were subcutaneously implanted in the flanks of Nu/Nu nude mice. When the length and width of the tumors reached 3 × 3 mm, mice were randomized in groups of five animals and treated with irrelevant (from control group, black-filled circles) or vaccination-induced PAbs (gray-lined open circles) obtained in rabbits (1 mg of total IgG/mouse/injection for both treatment groups) every 3 days. Tumor volumes corresponding to the kinetic growth of **(A)** H292 and **(B)** PC9ER tumors are shown. Data are means ± SD from five mice per group. Statistical analysis of tumor volume kinetics was performed using Mann–Whitney *U* test. *p < 0.05.

It was also desired to assess the influence of different mutations that favor resistance to HER1-targeting therapies on such impact. Cytotoxicity was compared in a panel of four NSCLC cell lines overexpressing mutated variants of this receptor. In addition to the above-mentioned H1975 and PC9ER, we included H3255 cells harboring L835R mutation that confers sensitivity to erlotinib, and PC9 cells expressing the exon 19 deletion. In the case of erlotinib-responsive H3255 cells, both PAbs and the combination of high-affinity cetuximab and trastuzumab were able to inhibit cell viability (**Figure 4C**). Alternatively, in H1975 (**Figure 4D**)-, PC9 (**Figure 4E**)-, or PC9ER (**Figure 4F**)-expressing mutations that render cells insensitive to gefitinib/erlotinib, only PAbs induced a significant decrease in cell viability. As observed, the MAbs were not cytotoxic in these models, neither alone nor combined. In PC9ER tumor cells, the dose increase in PAbs, and not the combination of cetuximab and trastuzumab, increased the magnitude of this effect (**Figure S7B**). Moreover, when PAbs obtained from immunized rabbits (selected to avoid complement-mediated cell cytotoxicity) were passively transferred to nude mice bearing H292-derived tumors (sensitive to TKIs and MAbs, **Figure 5A**) or more aggressive PC9ER-derived tumors (erlotinib-resistant, **Figure 5B**), which are also less sensitive to the combination of cetuximab and trastuzumab *in vivo* (Romaniello et al., 2020), they were able to significantly reduce tumor growth, in comparison to irrelevant PAbs obtained from a non-immunized control group.

Finally, in addition to cells lines representative of acquired resistance to the first generation of TKIs, we aimed to compare the cytotoxicity of the PAbs with the combinations of MAbs in tumor cells where the sensitivity to cetuximab was decreased after chronic treatment with this antibody. To this aim, H125 lung tumor cells overexpressing wild-type HER1 (29), which are equally sensitive to the PAbs and cetuximab plus trastuzumab or nimotuzumab plus trastuzumab combinations (**Figure S8A**), were grown with sustained exposure to increasing concentrations of cetuximab for 4.5 months. Then, we confirmed the induction of tolerance to high concentrations of this antibody in the resultant cells (H125CT) (**Figures S8B, C**). Unlike parental H125 cells, H125CT cells were insensitive to both combinations of MAbs, though the cytotoxicity of the TKI control suggested that these cells could be disturbed by robust inhibition of HER1 (Figure **S8D**). In this scenario, a significant reduction of the cell viability was also induced by the PAbs, further suggesting differential cytotoxicity as a result of HER1 and HER2 inhibition from a polyclonal or monoclonal perspective, in the context of lung tumor cells unresponsive to HER1-targeting therapies.

## 4 Discussion

Combinations of passive therapies targeting HER1 and HER2 oncogenes have arisen as an attractive alternative in cancer immunotherapy, proving to be effective in controlling tumor burden (21) and preventing the development of resistance to TKI and MAbs (40, 42). Additional studies have suggested that mixtures of antibodies targeting non-overlapping epitopes are more efficient than single antibodies in inducing HER receptors’ downregulation (43) and inhibiting tumor progression (44, 45). Our proposal relies on eliciting endogenous antibodies by immunization with the HER1+HER2/VSSP vaccine candidate (23). The main goal of this study was to compare vaccination-induced PAbs with dual combinations of registered MAbs of the same specificity, with emphasis on their ability to prompt the Fab-dependent effector mechanisms.

PAbs recognize at least three subdomains on each receptor (I, III, and IV) preventing the binding of low- and high-affinity MAbs. The lack of an adequate subdomain II expression and folding for both receptors limited the direct demonstration of its recognition by the PAbs. However, we detected the inhibition of the interaction between HER2 and pertuzumab, which recognizes an epitope located in this subdomain (8), although the possibility of steric hindrance mediated by the PAbs in recognizing adjacent regions cannot be ruled out. Then, the capacity of the PAbs to recognize HER1/HER2-subdomain II must be evaluated in further studies.

The interaction of the PAbs with multiple domains in the targets could explain some of their functional attributes. Recognition of non-overlapping epitopes in the targets could promote extensive receptor cross-linking, lattice formation at the cell surface, and detection of receptor–PAbs complexes by the endocytic machinery (45, 46) explaining PAbs-mediated endocytosis and degradation of the targets, even in the presence of mutations in HER1. This result is relevant considering that defective ubiquitination and endocytosis of the receptor characterizes some HER1 mutants (32). Moreover, PAbs-induced degradation of HER2 could also disrupt its mediated stabilization of HER1, and further contribute to the loss of HER1 expression.

Upon stimulation with specific ligands, a structural rearrangement approximates subdomains I and III of HER1 forming the ligand-binding pocket and exposing the dimerization arm (47). Hence, recognition of these subdomains by the PAbs explains inhibition of ligand-mediated receptor phosphorylation. Receptor degradation mediated by the PAbs, along with inhibition of their phosphorylation, was translated into an enhanced impairment of cell viability regarding MAbs combinations in tumor lines overexpressing wild-type or mutated variants of HER1, as well as cetuximab-tolerant cells. In addition, vaccination-induced PAbs were able to reduce the growth of tumor models representative of both sensitivity and resistance to HER1-targeting therapies. Overcoming resistance to first-generation TKIs has been previously achieved in preclinical studies using the cetuximab plus trastuzumab mixture, but their combination with TKIs (40) or with another MAb targeting HER3 (41) might enhance their toxicity in the clinical setting, for which endogenous PAbs induced by vaccination could be a safe alternative. Since the present study focused on Fab-mediated mechanisms of action, these *in vivo* studies were conducted using PAbs recovered from rabbits immunized following the same schedule as with mice. Nevertheless, it would be equally interesting to compare the contribution of the immune system to the overall antitumor effect of the PAbs (and MAbs combinations) evaluated. To this aim, upcoming studies might compare the antitumor effect, as well as the induction of antibody-dependent cellular cytotoxicity (ADCC) and further activation of DC through NK cells activated by the PAbs or the murine precursors of nimotuzumab (R3), cetuximab (C225), and trastuzumab (4D5) MAbs *in vivo*, to complement the Fab-associated mechanisms characterized in our study.

Alternatively, the ability of the PAbs to recognize subdomains III and IV suggests that they could be effective in the context of HER1-ECD mutations like EGFRvIII, which lacks most of the subdomains I and II (48) and signals constitutively (49). Likewise, recognition of multiple epitopes in the targets might overcome HER1 somatic mutations like G465R, G465E, S468R, and S492R that prevent MAbs binding (48) or HER2 mutations like L755S, V842I, and K753I, which predict resistance to trastuzumab or lapatinib (50). Then, it would be relevant to address these hypotheses in future studies.

It is worth mentioning that, despite the encouraging results obtained, the efficacy of cancer vaccines has been rather limited to date (51). Challenges in cancer vaccine development include (1) the immunosuppressive microenvironment of established disease, (2) immune system exhaustion in patients, and (3) low antigen immunogenicity (51). The potential of our candidate could rely on antigens and adjuvant selection. Inhibition of two major oncogenes could be advantageous over monovalent vaccines, or vaccines based on less relevant antigens. Also, immunogenic proteoliposomes (VSSP) included as adjuvant stimulate strong antigen presentation and CTL functionality (52), allowing avoidance of tolerance to self-antigens like HER1 and VEGF in preclinical and clinical settings (53–55).

Because of the selection of VSSP as adjuvant in our vaccine candidate, we place interest in the scenario of tumors that lack or have few tumor-infiltrating lymphocytes (defined as immunologically cold tumors), characterized by an immunosuppressive microenvironment. Cancer vaccines are suggested to strengthen T-cell expansion and infiltration in this tumor type (56). In addition, VSSP has proven to be able to reduce the frequency and functionality of MDSCs (57). Furthermore, HER1 inhibition has been connected to the downregulation of immunosuppressive molecules like PD-L1 (58). Nevertheless, increasing reports claim that cancer vaccines must be administered in combination with other therapies (59). Then, the HER1+HER2/VSSP vaccine could be used in combination with drugs to restore the immune response like TGF-β-targeting MAbs, adenosine inhibitors, or immunomodulators like TLR agonists to enhance its therapeutic efficacy.

Finally, in the present study, no evidence of tissue damage was found after histopathological analysis of organs from immunized mice, suggesting the lack of toxicity in this model (**Figure S9**). Previous studies on non-human primates and castration-resistant prostatic carcinoma patients immunized with the HER1 vaccine demonstrated its immunogenicity without evidence of severe adverse events (53, 60). Still, the proposed vaccine candidate adds HER2 as an antigen and trastuzumab-like antibodies can be detected within the PAbs (**Figure 1D**), which might raise concerns regarding cardiotoxicity (61). Hence, the safety of the proposed vaccine candidate remains a question that should be elucidated in relevant preclinical models and the clinic.

In summary, our study proposes the generation of endogenous PAbs targeting HER1 and HER2 by immunization with a vaccine candidate to impair tumors expressing these receptors where registered MAbs are not effective, or where its administration is precluded by elevated toxicity. Even though the complexity of tumor progression and resistance emergence in patients cannot be accurately represented in simpler models, complementation of these findings with other scenarios of resistance to antitumor therapies will benefit the generation of additional evidence that could be tested in the clinical setting.

## Data availability statement

The original contributions presented in the study are included in the article/**Supplementary Material**. Further inquiries can be directed to the corresponding author.

## Ethics statement

This study was reviewed and approved by Center of Molecular Immunology’s Institutional Animal Care and Use Committee.

## Author contributions

GB-B, NGS, YY and BS-R conceptualized and designed the study. GB-B, LG, DP-M, DH-F, TF-B, GD-O and ML contributed with the development of the experiments. AR-C conducted animal studies. GB-B, DP-M and TF-B processed the data, and performed statistical analysis. GB-B, YY and BS-R analyzed and interpreted the data. GB-B and BS-R drafted the manuscript. NGS, YY and BS-R revised the manuscript. All authors contributed to the article and approved the submitted version.

## Funding

This study was supported by the IUBMB Wood Whelan Research Fellowship, the Weizmann Institute of Sciences and the Center of Molecular Immunology.

## Acknowledgments

The authors would like to acknowledge Dr. Mary Luz Uribe Ríos for assessment during immunofluorescence assay execution and analysis, and Dr. Addys González Palomo for expert interpretation of the histological analysis. The authors are extremely thankful to Dr. Victor Brito Navarro for his support with statistical analysis and careful revision of the manuscript.

## Conflict of interest

The authors declare that the research was conducted in the absence of any commercial or financial relationships that could be construed as a potential conflict of interest.

## Publisher’s note

All claims expressed in this article are solely those of the authors and do not necessarily represent those of their affiliated organizations, or those of the publisher, the editors and the reviewers. Any product that may be evaluated in this article, or claim that may be made by its manufacturer, is not guaranteed or endorsed by the publisher.

## Abbreviations

ADCC, antibody-dependent cellular cytotoxicity; AREG, amphiregulin; CTL, cytotoxic T lymphocytes; DC, dendritic cells; ECD, extracellular domain; EGF, epidermal growth factor; EREG, epiregulin; ERK1/2, extracellular signal-regulated kinase 1/2; HB-EGF, heparin-binding EGF-like growth factor; HER1 and HER2, human epidermal growth factor receptor 1 and 2; KRas, Kirsten rat sarcoma virus; MAbs, monoclonal antibodies; MDSC, myeloid-derived suppressor cells; NK, natural killer; NSCLC, non-small cell lung cancer; PAbs, polyclonal antibodies; PD-L1, programmed death-ligand 1; SD, standard deviation; STAT3, signal transducer and activator of transcription 3; TLR, toll-like receptor; TKIs, tyrosine kinase inhibitor; PI3K, phosphoinositol kinase 3; VSSP, very small-sized proteoliposomes.
