# Supplementary figures and images for: Polyclonal antibody-induced downregulation of HER1/EGFR and HER2 surpasses the effect of combinations of specific registered antibodies
Source: Front Oncol. 2022 Nov 2;12:951267. doi: 10.3389/fonc.2022.951267 (PMC9667895; doi:10.3389/fonc.2022.951267)

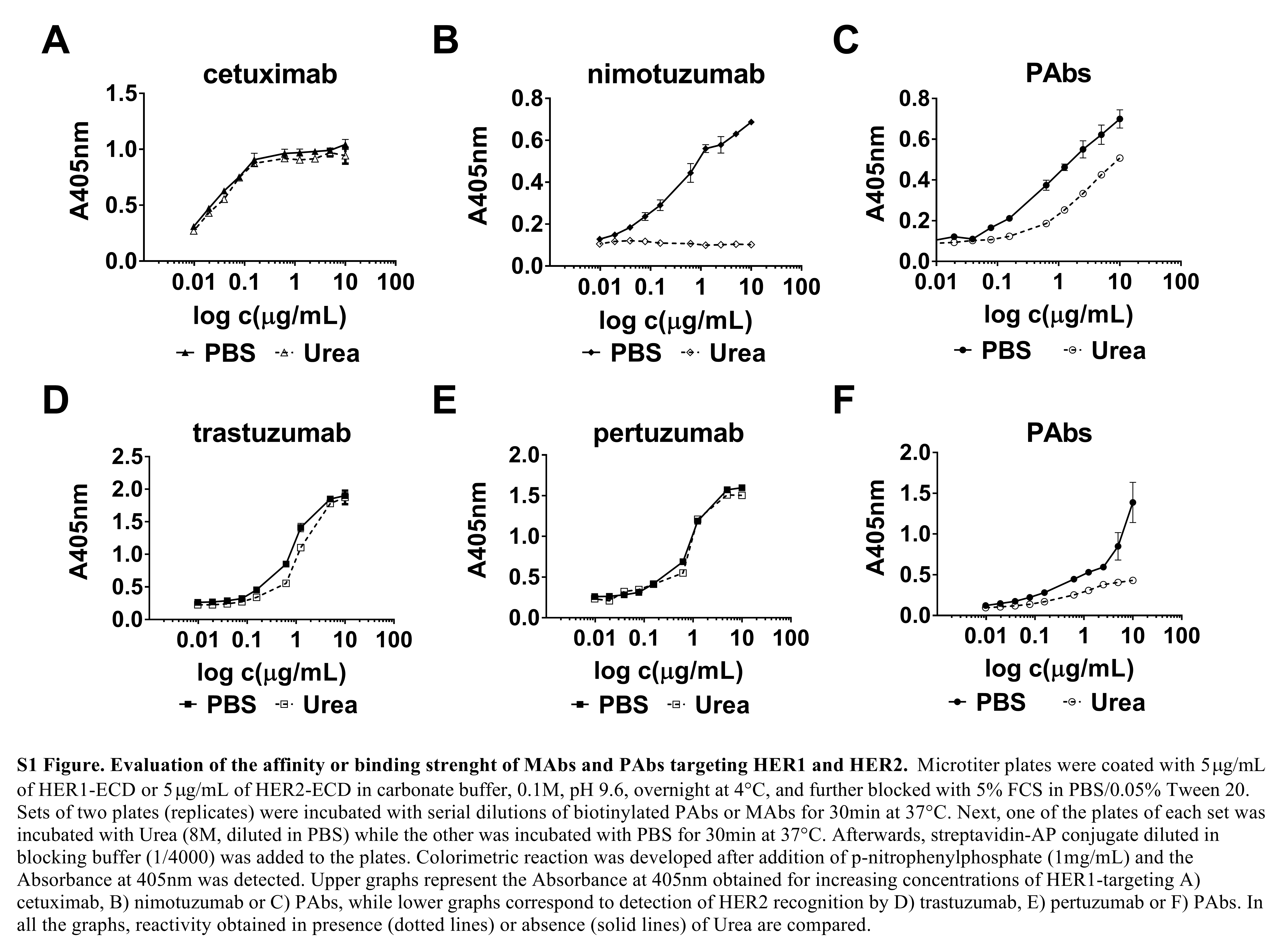

Supplement: Supplementary file 1 [file Image_1.tif]

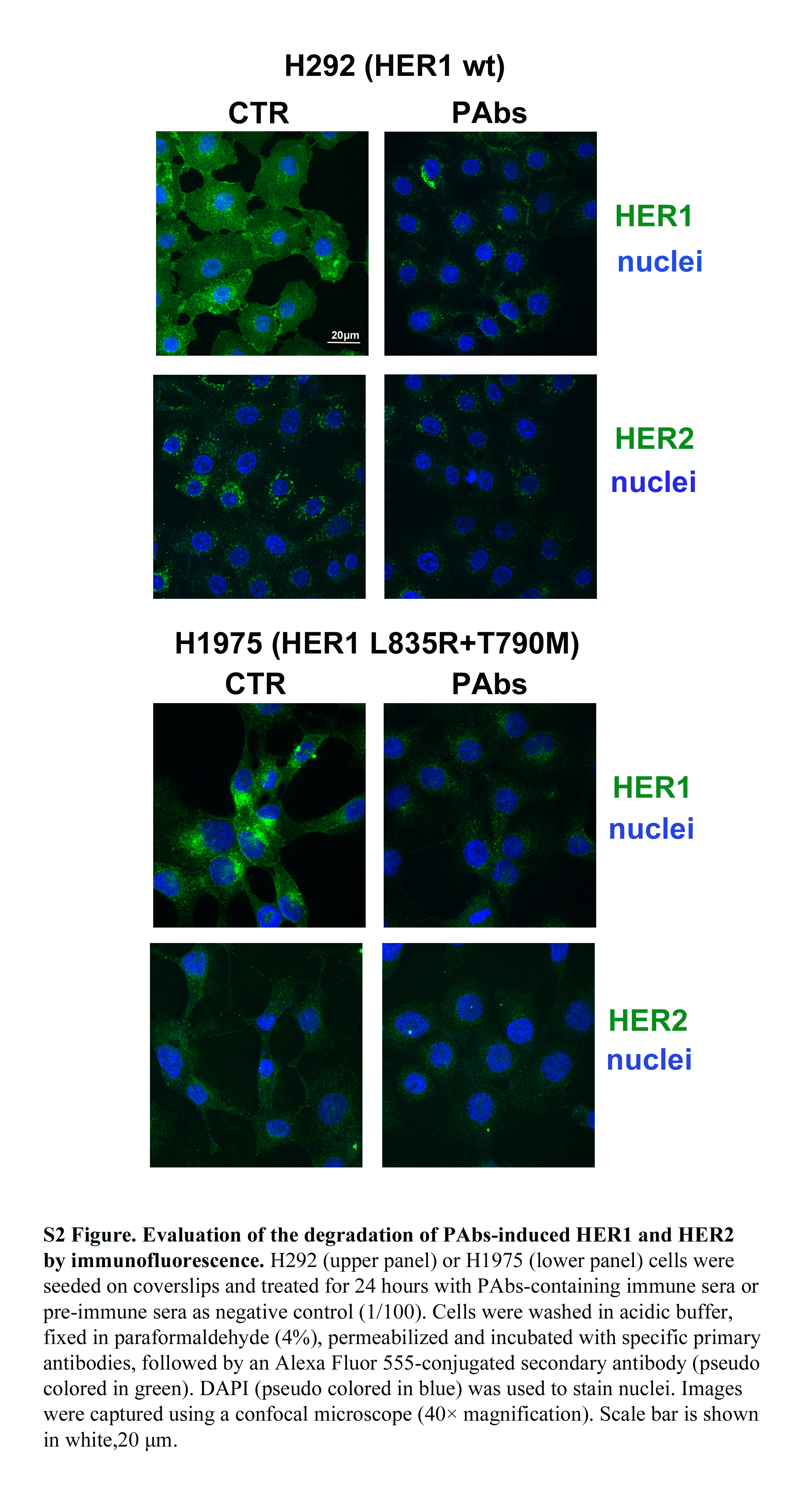

Supplement: Supplementary file 2 [file Image_2.tif]

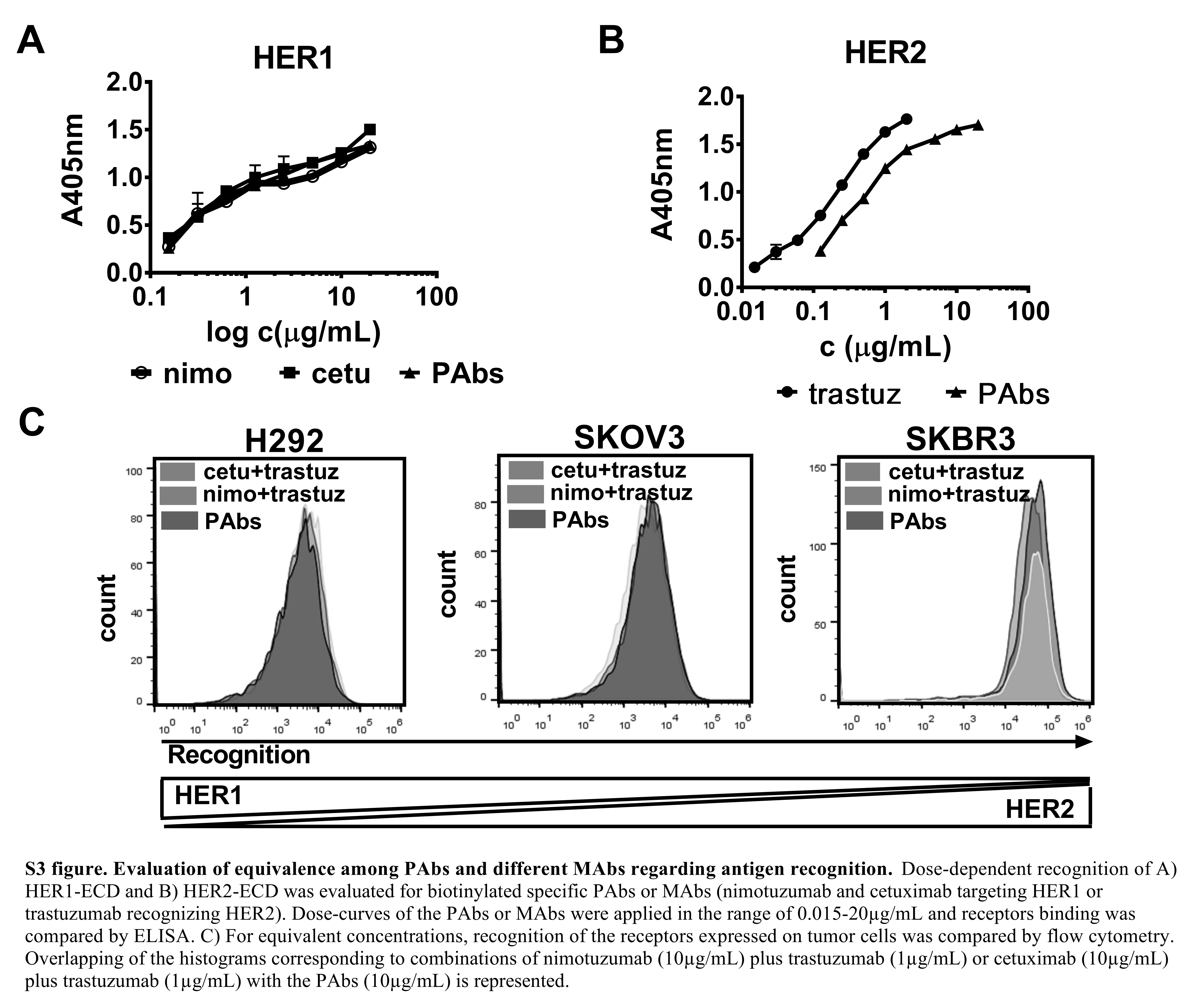

Supplement: Supplementary file 3 [file Image_3.tif]

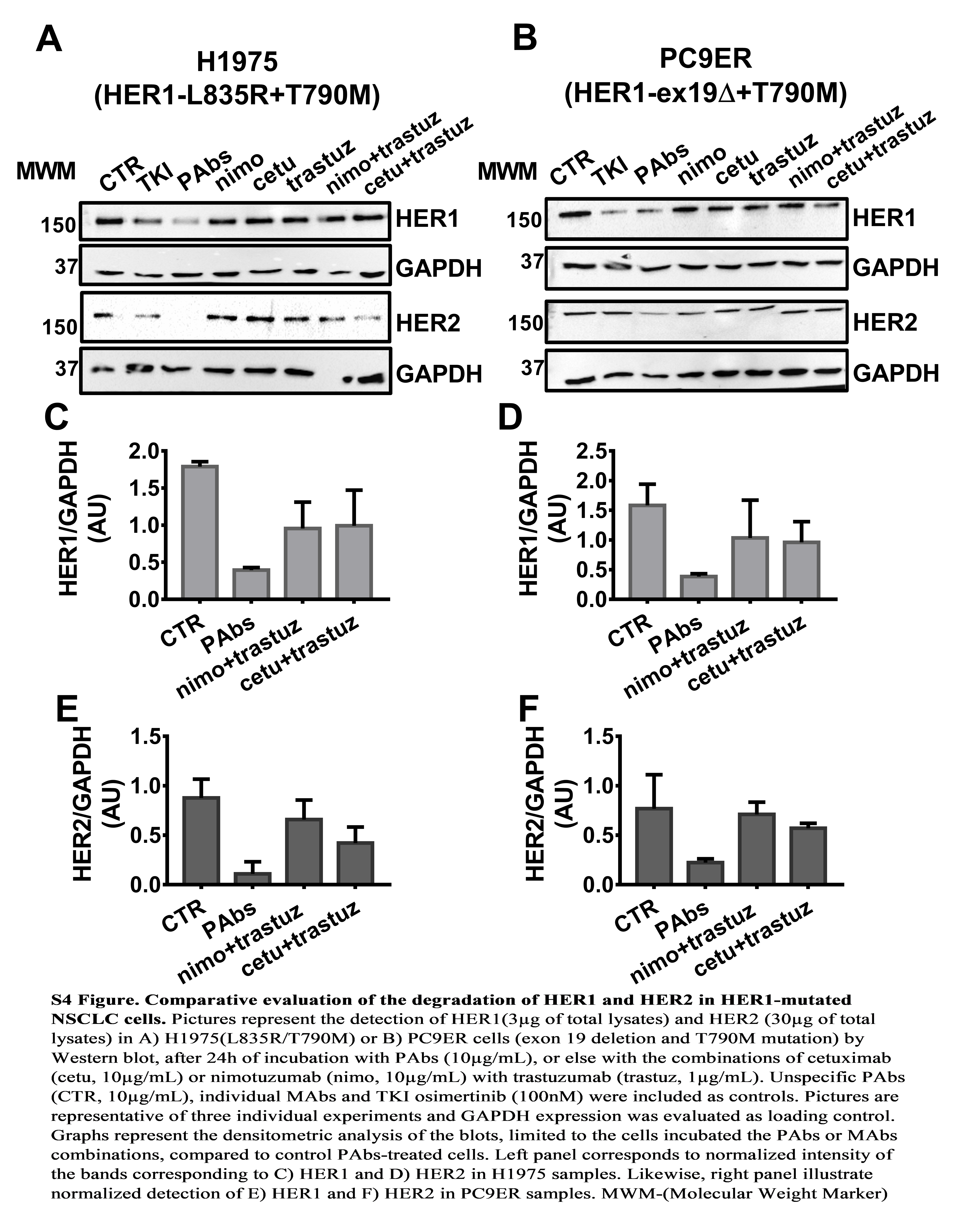

Supplement: Supplementary file 4 [file Image_4.tif]

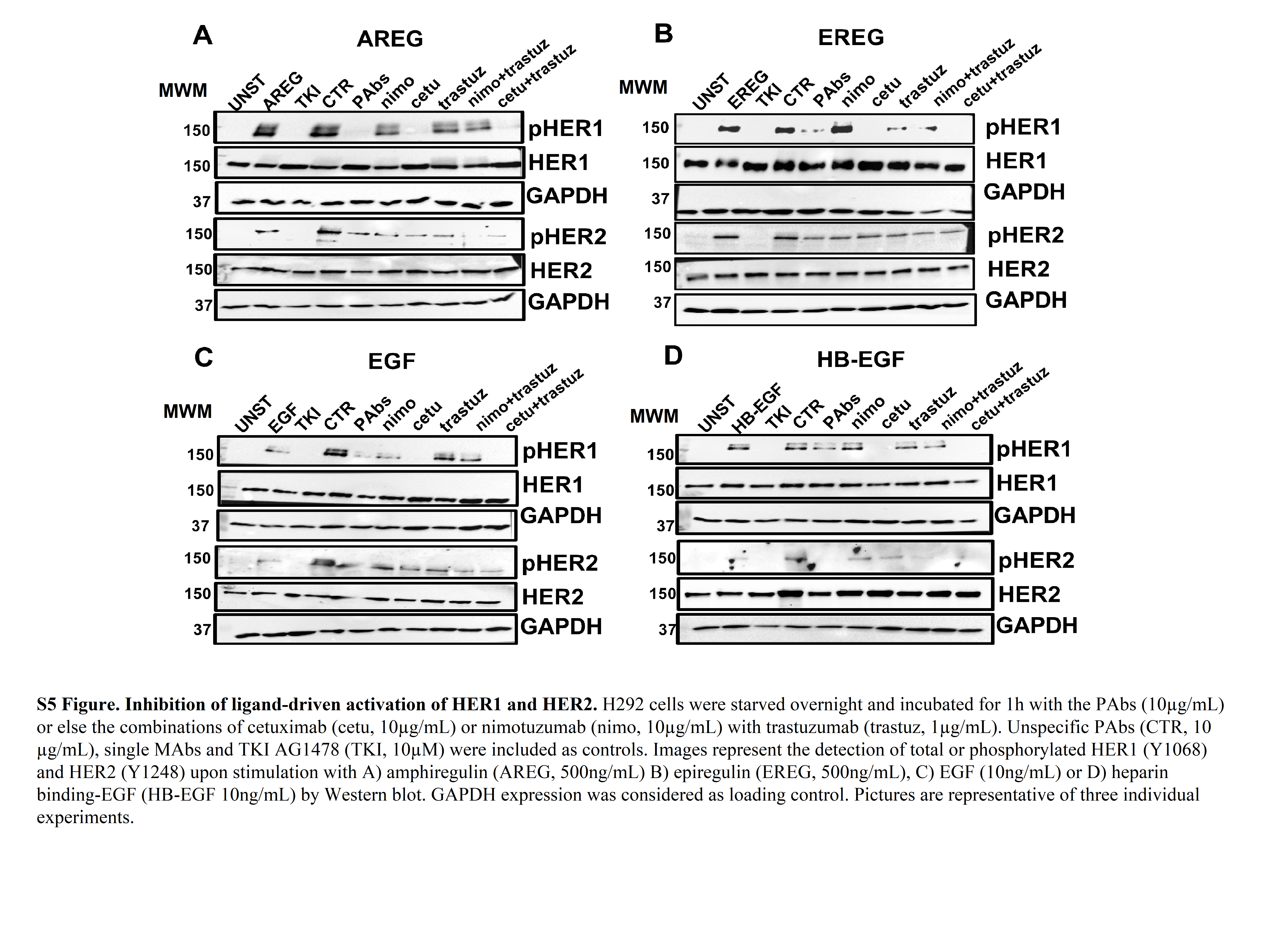

Supplement: Supplementary file 5 [file Image_5.tif]

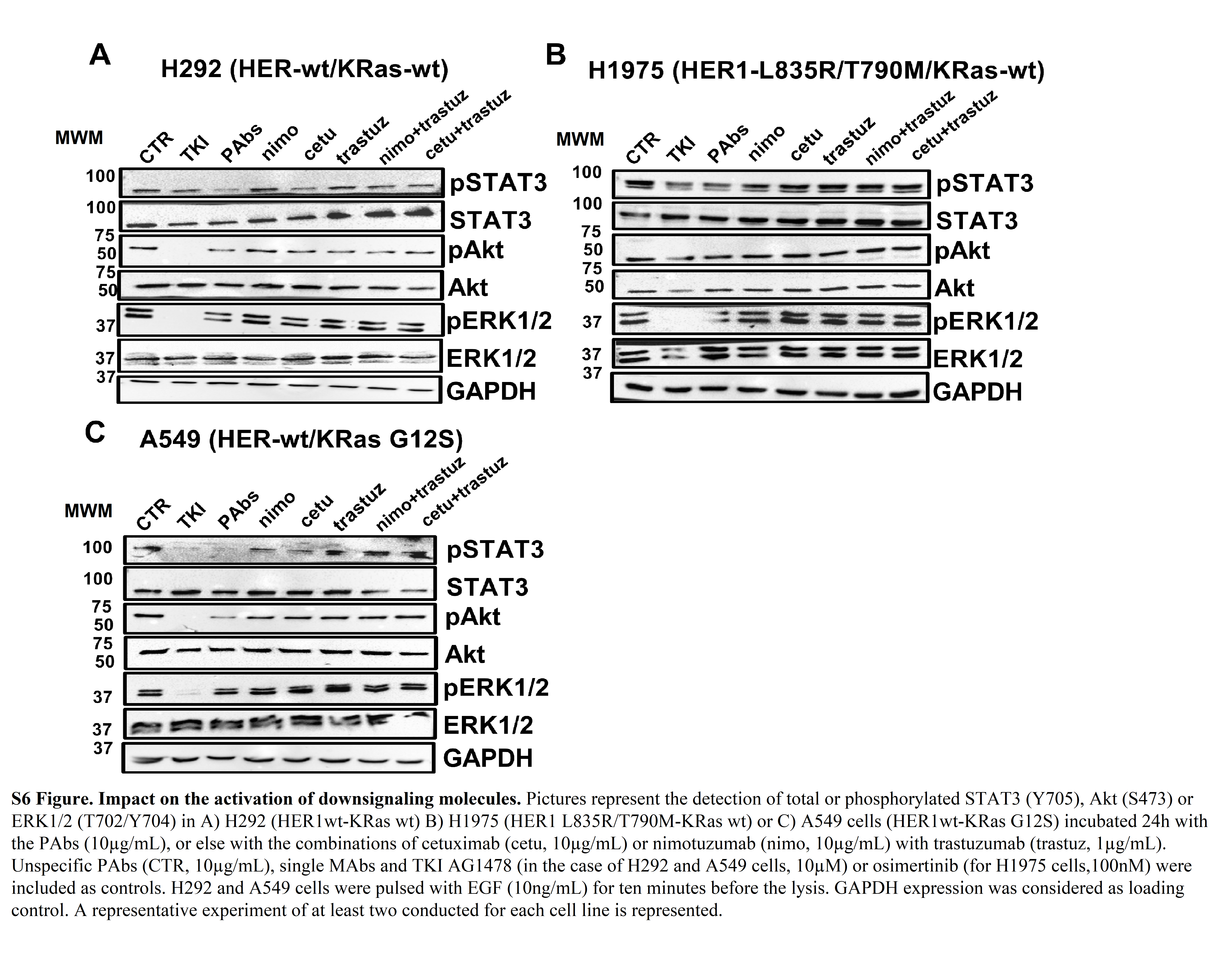

Supplement: Supplementary file 6 [file Image_6.tif]

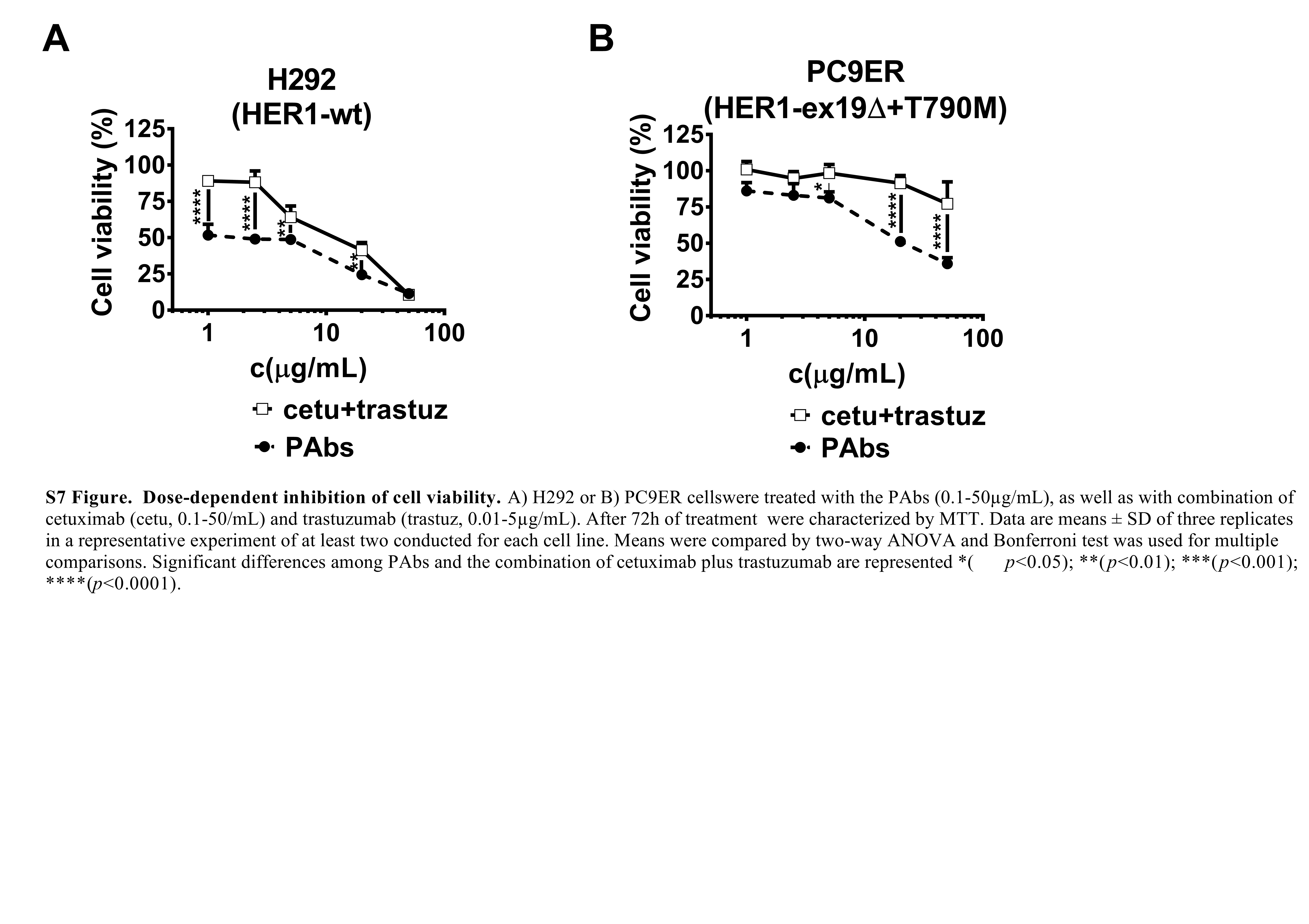

Supplement: Supplementary file 7 [file Image_7.tif]

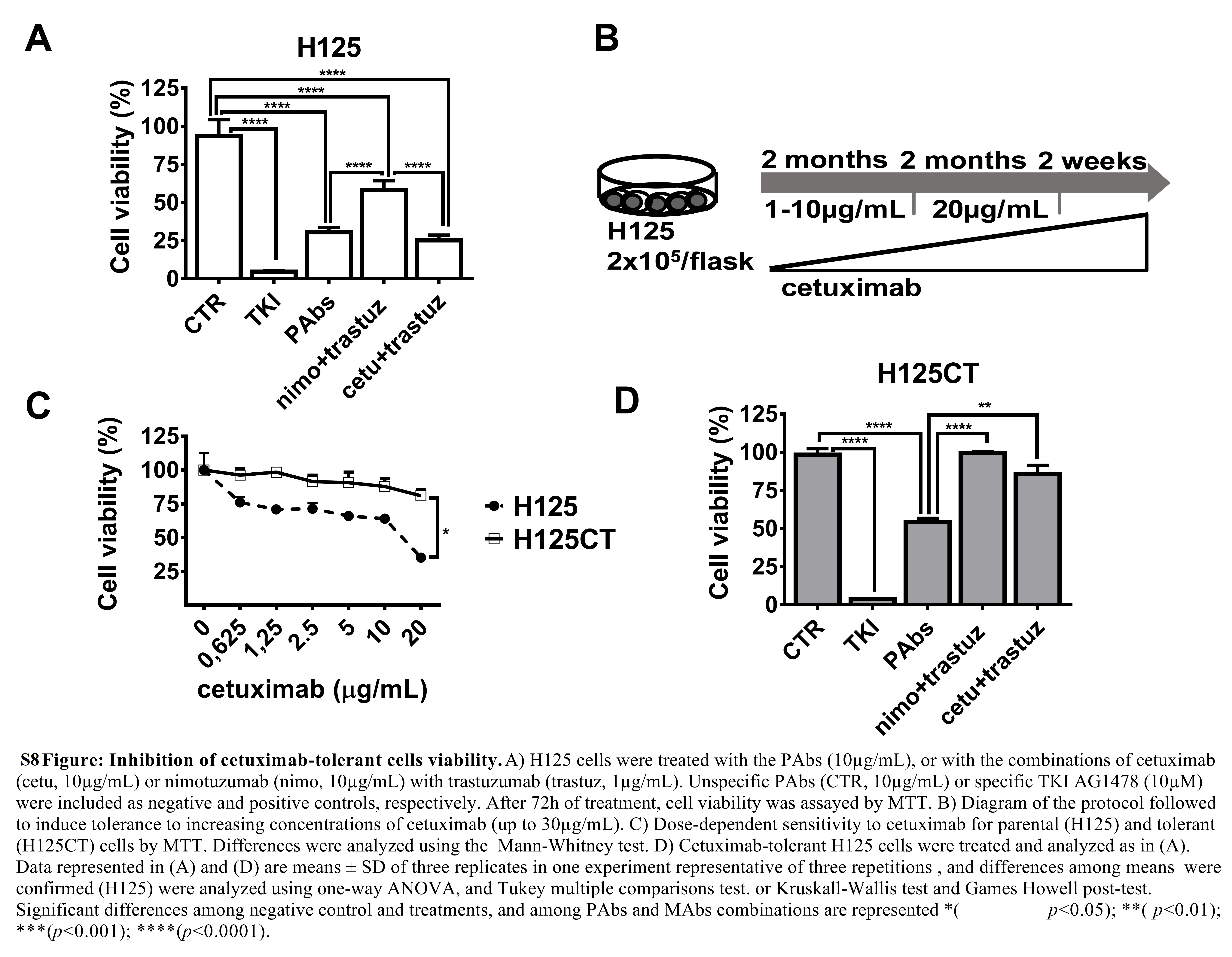

Supplement: Supplementary file 8 [file Image_8.tif]

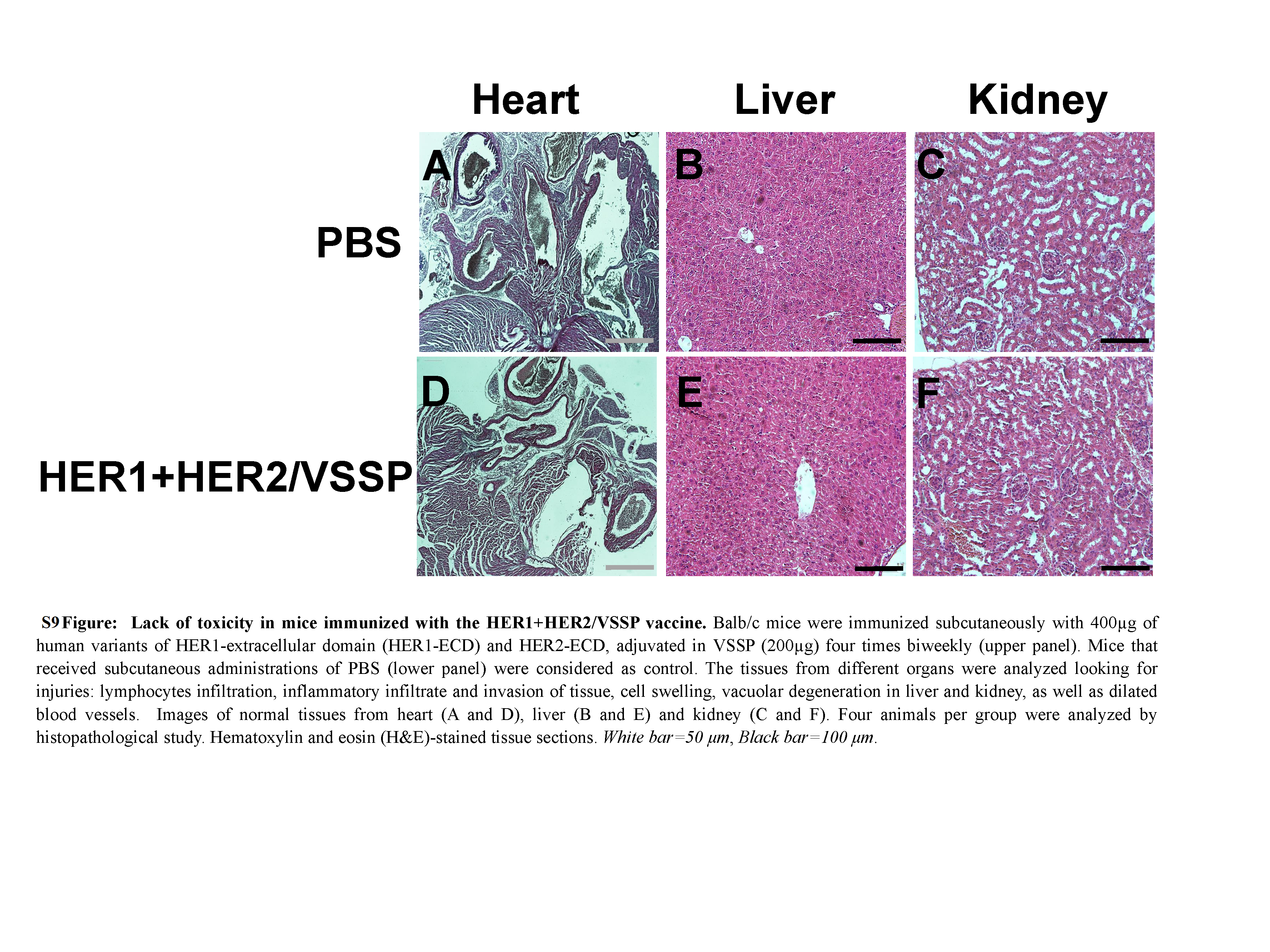

Supplement: Supplementary file 9 [file Image_9.tiff]
